# Supplementary material for: Assessing disease experience across the life span for individuals with osteogenesis imperfecta: challenges and opportunities for patient-reported outcomes (PROs) measurement: a pilot study
Source: Orphanet J Rare Dis. 2019 Jan 29;14:23. doi: 10.1186/s13023-019-1004-x (PMC6350324; doi:10.1186/s13023-019-1004-x)
Supplement: Supplementary file 1 — Questionnaire was developed by the study team and used to gather specific data points for this study regarding demographics and patient history. (PDF 82 kb) [file 13023_2019_1004_MOESM1_ESM.pdf]

Information About You

When answering these questions, "you" means either you or your child. If you have multiple children, please only answer the questions for one child.

Today's Date: __ __ / __ __ __ / __ __ __ __ (DAY / MONTH / YEAR)

Date of Birth: __ __ / __ __ __ / __ __ __ __ (DAY / MONTH / YEAR)

Current Age: ____________ Years [CALCULATED FIELD]

Gender: ○ Male ○ Female ○ Unknown

Race *(check all that apply):*

- ☐ American Indian or Alaska Native
- ☐ Asian
- ☐ Black or African American
- ☐ Native Hawaiian or Other Pacific Islander
- ☐ White
- ☐ Unknown
- ☐ Refused

Ethnicity (*select one):*

- ○ Hispanic, Latino, or Spanish Origin
- ○ Not Hispanic, Latino, or Spanish Origin
- ○ Unknown
- ○ Refused

Do you know what type of Osteogenesis Imperfecta you have? ○ Yes ○ No ○ Don't Know

Please indicate what type of Osteogenesis Imperfecta you have:

- ○ I - Type 1
- ○ II - Type 2
- ○ III - Type 3
- ○ IV - Type 4
- ○ V - Type 5
- ○ Other Please specify:
- ○ VI - Type 6
- ○ VII - Type 7
- ○ VIII - Type 8
- ○ IX - Type 9
- ○ Bruck Syndrome

Do you know how your Osteogenesis Imperfecta was diagnosed? ○ Yes ○ No ○ Don't Know ○ Not Diagnosed

How was your Osteogenesis Imperfecta diagnosed? (*Select all that apply*)

- ☐ Skin Biopsy/collagen studies
- ☐ Blood/DNA studies
- ☐ Clinical history and radiographs highly suggestive of Osteogenesis Imperfecta

Do other members of your family have Osteogenesis Imperfecta? (Please consider family members who are no longer living.)

○ Yes ○ No ○ Don't Know

Please indicate which members of your family have Osteogenesis Imperfecta (Please include family members who are no longer living): (*Select all that apply*)

- ☐ Mother
- ☐ Father
- ☐ Sister(s)
- ☐ Brother(s)
- ☐ Aunt(s)
- ☐ Uncle(s)
- ☐ Cousin(s)
- ☐ Niece(s)
- ☐ Nephew(s)
- ☐ Granddaughter(s)
- ☐ Grandson(s)
- ☐ Mother's mother
- ☐ Mother's father
- ☐ Father's mother

Information About You

When answering these questions, "you" means either you or your child. If you have multiple children, please only answer the questions for one child.

- ☐ Daughter(s)
- ☐ Son(s)
- ☐ Father's father

What is your means of mobility? (*Select all that apply*)

- ☐ Walk unaided
- ☐ Walk with crutches or walker
- ☐ Walk with leg braces
- ☐ Walk with cane
- ☐ Manual wheelchair
- ☐ Other Please specify: ______________________________
- ☐ Electric wheelchair
- ☐ Use wheelchair outside the home only
- ☐ Use service dog
- ☐ Use service dog outside the home only
- ☐ Not mobile

Does your means of mobility allow you the level of independence that you desire? ○ Yes ○ No ○ Don't Know

How tall are you? (inches): ______________

Did you have fractures before birth?		○ Yes	○ No	○ Don't Know
Age at time of first fracture? (If pre-natal or birth, enter 0)	________	○ Years	○ Months	○ Weeks ☐ Don't Know
Age at time of most recent fracture?	________	○ Years	○ Months	○ Weeks ☐ Don't Know

Approximately how many fractures have you had in your lifetime?	________	☐ NA	☐ Don't Know
Approximately how many fractures you had from age 0-10 years old?	________	☐ NA	☐ Don't Know
Approximately how many fractures you had from age 11-19 years old?	________	☐ NA	☐ Don't Know
Approximately how many fractures you had from age 20-49 years old?	________	☐ NA	☐ Don't Know
Approximately how many fractures did you have after the age of 50?	________	☐ NA	☐ Don't Know

Have you ever had rodding surgery of your arms or legs	○ Yes	○ No	○ Don't Know
Have you ever had spine surgery	○ Yes	○ No	○ Don't Know
Have you ever had chronic or recurrent lung or breathing problems?	○ Yes	○ No	○ Don't Know
Do you have hearing loss?	○ Yes	○ No	○ Don't Know
Do you wear a hearing aid?	○ Yes	○ No	○ Don't Know
Do you have a Cochlear implant?	○ Yes	○ No	○ Don't Know
Have you had a stapedectomy?	○ Yes	○ No	○ Don't Know

Date of most recent hearing exam __ __/__ __ __/__ __ __ __ (DAY / MONTH / YEAR) ○ Don't Know

Have you ever been pregnant? ○ Yes ○ No ○ Not Applicable

How many times have you been pregnant? __________

How many times did you deliver (live births?) __________

In the last seven days, have you been on any medications, treatments, and/or therapies for the following?

Anxiety	○ Yes	○ No	○ Don't Know
Depression	○ Yes	○ No	○ Don't Know
Fatigue	○ Yes	○ No	○ Don't Know
Pain	○ Yes	○ No	○ Don't Know
Sleep Disturbances	○ Yes	○ No	○ Don't Know

What medications did you take in the last 7 days? ______________________________

Do the last seven days represent a typical 7 days for you for the following?

Anxiety	○ Yes	○ No	○ Don't Know	Why not? __________
Depression	○ Yes	○ No	○ Don't Know	Why not? __________

Information About You

When answering these questions, "you" means either you or your child. If you have multiple children, please only answer the questions for one child.

Fatigue	○	Yes	○	No	○	Don't Know	Why not?	___________
Pain	○	Yes	○	No	○	Don't Know	Why not?	___________
Sleep Disturbances	○	Yes	○	No	○	Don't Know	Why not?	___________
